# Identification of a major *rif *transcript common to gametocytes and sporozoites of *Plasmodium falciparum*

**DOI:** 10.1186/1475-2875-9-147

**Published:** 2010-05-28

**Authors:** Christian W Wang, Steven B Mwakalinga, Colin J Sutherland, Samana Schwank, Sarah Sharp, Cornelus C Hermsen, Robert W Sauerwein, Thor G Theander, Thomas Lavstsen

**Affiliations:** 1 Department of International Health, Immunology, and Microbiology, University of Copenhagen, Copenhagen, Denmark; 2 Department of Infectious Diseases, Copenhagen University Hospital (Rigshospitalet), Copenhagen, Denmark; 3 Department of Infectious and Tropical Diseases, Immunology Unit, London School of Hygiene and Tropical Medicine, London, UK; 4 Department of Medical Microbiology, Radboud University Nijmegen Medical Centre, Nijmegen, the Netherlands

## Abstract

**Background:**

The *Plasmodium falciparum *parasite is transmitted in its sexual gametocyte stage from man to mosquito and as asexual sporozoites from mosquito to man. Developing gametocytes sequester preferentially in the bone marrow, but mature stage gametocytes are released to the bloodstream. Sexual stage parasite surface proteins are of interest as candidate target antigens for transmission blocking vaccines.

**Methods:**

In this study, the transcript profiles of *rif *and *var *genes, known to encode surface antigens in asexual blood stage parasites, were investigated at different stages of 3D7/NF54 gametocytogenesis and in sporozoites.

**Results:**

Gametocytes exhibited a *rif *transcript profile unlinked to the *rif *and *var *transcript profile of the asexual progenitors. At stage V, mature gametocytes produced high levels of a single *rif *gene, PF13_0006, which also dominated the *rif *transcript profile of sporozoites. All *var *genes appeared to be silenced in sporozoites.

**Conclusions:**

The most prominent variant surface antigen transcribed in both gametocytes and sporozoites of 3D7/NF54 is a single variant of the RIFIN protein family. This discovery may lead to the identification of the parasites binding ligands responsible for the adhesion during sexual stages and potentially to novel vaccine candidates.

## Background

*Plasmodium falciparum *is transmitted from infected humans to mosquitoes via sexual stages called gametocytes. Immature gametocytes arise from erythrocytic asexual stages and sequester preferentially in the bone marrow [1] for about 9-12 days before reaching maturity and emerging into peripheral blood [2]. Unlike other species of malaria parasites, five different stages of *P. falciparum *gametocyte development have been described *in vitro *by morphological criteria [2], known as stages I to V. Only at stage V are gametocytes released to the bloodstream and become infectious to mosquitoes of the *Anopheles sp*. A mean circulation time of 6.4 days with a range of 1.3-22.2 days has been reported [3], but aggregation of the mature stage V gametocyte in the sub-dermal capillaries has been proposed [4,5]. In the mosquito midgut, the male gametocyte transforms into eight motile microgametes and emerges from the erythrocyte within minutes (exflagellation), whereas the female emerges as a single round shaped macrogamete. Fertilization is required to initiate the mosquito stages of parasite development, resulting 10-18 days later in infectious sporozoites ready to invade a new human host.

It is thought that the sequestration during maturation of *P. falciparum *gametocytes allows avoidance of phagocytic clearance in the spleen, as immature sexual stages circulate in splenectomised hosts [6]. Several host receptors have been implicated in gametocyte adhesion, but at different stages of development. At stages I-IIA, CD36 seems to be the main receptor for adhesion [7-9], and at stages III-IV, ICAM-1, CD49c, CD166, and CD164 are candidate receptors [10]. Parasite ligands responsible for this sequestration are unknown, but identification of the relevant surface proteins is of great interest as they could be potential transmission blocking vaccine candidates [11,12].

Several multigene families encode proteins on the asexual parasite-infected erythrocytic surface such as PfEMP1, (*Plasmodium falciparum *erythocyte membrane protein 1), STEVOR (subtelomeric variable open reading frame) and RIFIN (repetitive interspersed family), and these are obvious candidates for studying gametocyte adhesion [8,9,13]. Hayward *et al *[8] found that the adhesion of early stage gametocytes to CD36 is mediated by PfEMP1, but not at later stages, where the knob structure is no longer present, and PfEMP1 seems to be confined to the parasite cytoplasm [7,8].

PfEMP1 s (200-400 kDa) are encoded by approximately 60 *var *genes per genome. The clonal variant proteins are known to mediate cytoadhesion of the infected erythrocyte to host receptors [14] and are associated with development of immunity to asexual stages and protection from severe disease [15,16]. Based on sequence analysis, *var *genes were grouped in five different types [17,18]. A study of *var *transcript abundances in gametocytes showed a degree of *var *transcription programming favouring a subset of type C genes in gametocytes unlinked to the phenotype of asexual progenitors and unlinked to the transcription of *stevor *genes [13].

RIFIN proteins are encoded by the two-exon *rif *gene family of interspersed repetitive DNA, predominantly located in the sub-telomeric regions. Each parasite genome encodes around 200 genes encoding proteins of 30-40 kDa. RIFINs have conserved amino and carboxy termini, and one to two predicted transmembrane domains delineating a proposed extracellular variable region. No function has yet been ascribed to RIFINs. It has been proposed that RIFIN variants belonging to two disctinct subgroups designated A- and B-type RIFINs [19], show different subcellular localization patterns. A-type RIFINs were found to be transported to the surface of the infected erythrocyte and B-type RIFINs were retained inside the parasitophorous vacuole [20]. Furthermore, Petter *et al *[21] using a semiquantitative cDNA cloning approach provided evidence for stage-specific transcriptional regulation patterns during gametocytogenesis for the two *rif*-types. Expression of these multigene families has to date not been systematically investigated in sporozoites.

In this study, variant-specific real-time amplification was used to provide the first quantitative transcriptional analysis of each member of the *rif *gene family in both *P. falciparum *gametocytes and in sporozoites. A single *rif *gene was found to dominate transcription in both sporozoites as in gametocytes.

## Methods

### Parasites

Parasites of *P. falciparum *3D7 and NF54 were cultured and induced to gametocytogenesis as previously described [13,22] at London School of Hygiene and Tropical Medicine, London, and at Radboud University Nijmegen Medical Centre, Nijmegen, respectively. RNA was taken from 3D7 gametocyte cultures on days 3, 5, and 10 and the gametocyte stages were determined as predominantly stage II, III, and V, respectively. RNA was taken from NF54 gametocyte culture at stage V. RNA was also taken from NF54 sporozoites isolated from the salivary glands of *Anopheles sp*. as previously described [23].

### RNA isolation and cDNA

Parasites were harvested in Trizol reagent (Invitrogen) and total RNA was extracted according to the manufacturer's instructions (Invitrogen). Samples were treated with DNase I (Sigma) to digest any genomic DNA and tested in real-time PCR for contamination, using a primer set for the *seryl-tRNA synthetase *gene, p90. RNA was reverse transcribed from random hexamers, using Superscript II (Invitrogen), according to the manufacturer's instructions (Invitrogen). RNA from previous studies stored at -80°C [13] was prepared for cDNA as described above. The RNA available was that taken from a 3D7 parasite line selected with IgG from semi-immune children, 3D7-Dodowa1, at the asexual ring stage and gametocyte stage III and from an unselected culture of clone 3D7 at the asexual ring stage, gametocyte stage II, III, and V.

### Primer design

The *rif *primer pairs described previously [24], *var *primer pairs, and primers to the endogenous control genes *seryl-tRNA synthetase *and *fructose-bisphosphate aldolase*, p61, described previously [25] with modifications [26] were used.

### Quantitative PCR

Quantitative PCR was executed on a Rotorgene RG-3000 thermal cycler (Corbett Research) applying QuantiTect SYBR Green PCR Master Mix (Qiagen) with primers at 20 μM, and internal control genes *seryl-tRNA synthetase *and *fructose-bisphosphate aldolase*, used for normalization as previously described [25]. Gene-specific standard curves were produced by determining the amplification efficiency relative to the single copy housekeeping gene, *seryl-tRNA synthetase*, based on quantitative measurements of 10-fold dilutions of genomic DNA and used to calculate the transcript copy number of each gene in tested cDNA.

## Results

### Rif gene transcript profiles in mature gametocytes and sporozoites is dominated by PF13_0006

**Figure 1:**
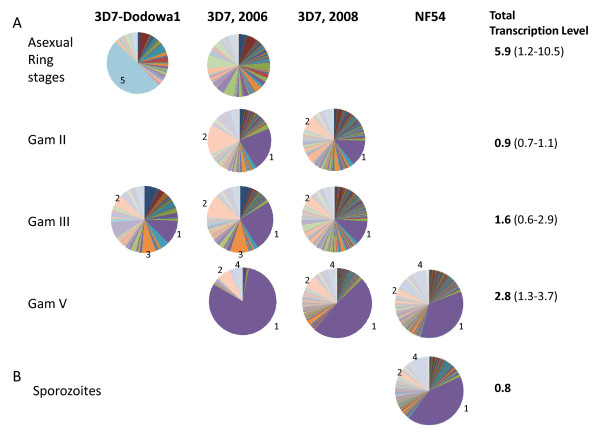
**Transcript abundances of *rif *genes**. (A) Transcript abundances of *rif *genes upon differentiation from asexual to sexual reproduction in a 3D7 parasite line selected with IgG from semi-immune children, 3D7-Dodowa1, an unselected culture of clone 3D7 (RNA from year 2006 and 2008), and an unselected NF54 parasite line. (B) Transcript abundances of *rif *genes in NF54 sporozoites. Transcript abundances were measured by quantitative PCR using a set of primers that amplify 154 *rif *genes from clone 3D7/NF54. Transcript abundance of each *rif *gene is shown as a proportion of the total transcript abundance of *rif *genes in a sample. Numbered sectors of the pie charts correspond to the following genes: 1, PF13_0006; 2, PFI0025c; 3, PFA0030c; 4, PFL0025c; 5, PFD1230c. Average transcript abundances are shown as the total transcript abundance of *rif *genes normalized to the average transcript abundance of endogenous control genes, *seryl-tRNA synthetase *and *fructose-bisphosphate aldolase*.

**Figure 2:**
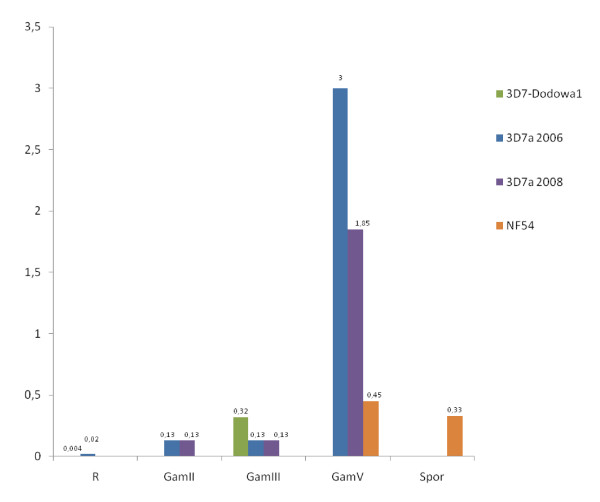
**Transcript abundance of PF13_0006 during differentiation from asexual to sexual reproduction and in sporozoites**. Transcript abundance of 1 corresponds to the mean level of two control genes, *seryl-tRNA synthetase *and *fructose-bisphosphate aldolase*. R, ring stage; GamII, gametocyte stage II; GamIII, gametocyte stage III; GamIV, gametocyte stage IV; GamV, gametocyte stage V; Spor, Sporozoites. The colour codes identify the parasite culture from which RNA was purified.

*Plasmodium falciparum *parasites were induced to develop into sexual gametocyte stages and the RNA was isolated for transcriptional analysis. 3D7 gametocytes were induced in three separate experiments to isolate stage II, III and V, whereas NF54 stage V gametocytes were available for one experiment. In gametocytes, *rif *transcript abundances increased as the parasites matured and transcripts of PF13_0006 and PFI0025c progressively dominated, with PF13_0006 being the most dominant at gametocyte stage V (Figure 1). The transcript abundance of PF13_0006 was more than 100 times higher at gametocyte stage V than during the asexual stages (Figure 2). PF13_0006 also dominated the *rif *transcription in sporozoites (Figure 1).

### The gametocyte rif transcript profile is unlinked to the phenotype of the asexual progenitors

Gametocyte stage parasites were induced from two different 3D7 lines. One unselected 3D7 and one 3D7 line selected with IgG from semi-immune children to select a variant surface antigen phenotype associated with severe malaria, 3D7-Dodowa1 [27]. The 3D7 and 3D7-Dodowa1 differed in their *var *transcript profiles at ring stage but showed similar *var *transcript profiles at gametocyte stages [13]. The *rif *transcript profiles of the two 3D7 lines also differed at ring stage parasites but appeared similar at the gametocyte stage III (Figure 1). RNA from later gametocyte stages of the selected line 3D7-Dodowa1 was not available.

### *Var *transcription in sporozoites

**Figure 3:**
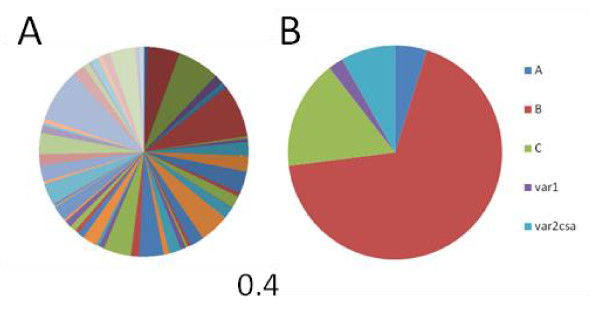
**Transcript abundances of *var *genes**. (A) Transcript abundances of each *var *gene shown as a proportion of the total transcript abundance of *var *genes in sporozoites of clone NF54. Transcript abundances were measured by quantitative PCR using a set of primers that amplify 59 *var *genes from clone 3D7/NF54. (B) Transcript abundances of each *var *gene shown as a proportion of the total transcript abundance of *var *genes and shown as proportion of *var *types according to the type described by Lavstsen *et al *[18]: A, B, C, *var1*, and *var2csa*. The total transcript abundance of *var *genes normalized to the average transcript abundance of endogenous control genes, *seryl-tRNA synthetase *and *fructose-bisphosphate aldolase *is shown. For comparison, asexual ring stage- and gametocyte stage V parasites showed total transcript abundances of 55 and 3, respectively [13].

The transcript abundance of *var *genes in sporozoites were investigated and showed that the transcript distribution was diverse and the levels were low (Figure 3) compared to those found in asexual ring stage- and gametocyte stage V parasites. Total transcript abundances of 0.4 (Figure 3), 55 and 3, respectively [13].

## Discussion

Antigens expressed on the surface of parasites and infected erythrocytes are attractive vaccine candidates. In this study, the transcription of variant gene families known to be expressed on the surface of infected erythrocytes was investigated. The data demonstrate a stage-specific transcription of *rif *genes in the developing gametocyte with up-regulation of a single *rif *gene: PF13_0006, in the mature stage V gametocytes. This result is supported by the study by Petter *et al *[21], who reported, using a plasmid clone counting approach, that the B1-type *rif *gene, PF13_0006 [19], but also PFI0025c, another B1-type *rif *gene dominated the *rif *gene transcript profile of developing stage II and III gametocytes. Here, we show that regardless of the *rif *and *var *transcript profile of the asexual progenitor, PF13_0006 dominates the transcript profile in gametocyte stages of both 3D7 and NF54 parasites lines. This implies that the encoded RIFIN has an important function in the developing gametocyte. However, expression studies of STEVOR in gametocytes have shown that the timing of protein expression and localisation may be uncoupled from that of transcription [28].

RIFINs could play a role in the sequestration of gametocytes, but the fact that the PF13_0006 transcript was maintained at high abundance in the non-adherent circulating stage V gametocytes makes it less likely that this protein is responsible for the sequestration. It is intriguing that PF13_0006 also dominated the *rif *transcript profile of sporozoites. It has previously been shown [24] that the transcription of PF13_0006 and PFI0025c during asexual blood stage development differs from that of most other *rif *genes in that they dominated the transcript profile in the late schizonts and in the early rings. The profile was unlinked to the *rif *transcript profile during the mid ring and early trophozoite stages and the VSA-phenotype of the parasite. This finding led to the suggestion that the proteins encoded by these two genes may be associated with the free merozoite stages. PF13_0006 may serve different functions depending on the developing pathway of the parasite; however, a common feature for extracellular gametes, sporozoites and merozoites is the need to "locate" another cell; sporozoites and merozoites in the bloodstream binding to hepatocytes and erythrocytes, respectively, and female and male gametes inside the mosquito midgut binding at fertilization. A role for RIFINs in these extracellular stages is at this point speculative but could include sensing, binding or both. However, localization of these RIFINs needs to be investigated, as surface expression on gametocytes, gametes, sporozoites, and merozoites has not as yet been established.

The transcription of *rif *genes is unlinked to *var *transcription in developing gametocytes and although PfEMP1 may have a role in sequestration of developing gametocytes to different host ligands, the function of RIFINs during gametocyte maturation remains to be confirmed.

## Conclusions

In conclusion, this study has demonstrated that the *rif *transcript profile of gametocytes is unlinked to that of asexual progenitors. In particular a single *rif *gene, PF13_0006, exhibits a consistently high transcript abundance in mature gametocyte stage V and in sporozoites. If homologues RIFINs of the B1-type are expressed on the surface of gametocytes, sporozoites, and merozoites of different origin this could lead to a possible vaccine candidate in all stages of the parasites life cycle.

## Competing interests

The authors declare that they have no competing interests.

## Authors' contributions

CWW carried out molecular biology studies, analysed data and wrote the paper. TL, SBM and TGT participated in the design, coordination and analysis of the study and helped to draft the manuscript. CJS, SS, and SS participated in the design and coordination of the gametocyte studies, helped analyse data and draft the manuscript. CCH and RWS participated in the design and coordination of the gametocyte and sporozoite studies, helped analyse data and draft the manuscript. All authors read and approved the final manuscript.

## References

[B1] Smalley ME Abdalla S Brown J The distribution of *Plasmodium **falciparum *gametocytes in the internal organs and peripheral circulation Trans R Soc Trop Med Hyg 1980 75 103 105 10.1016/0035-9203(81)90019-5 7022784

[B2] Hawking F Wilson ME Gammage K Evidence for cyclic development and short-lived maturity in the gametocytes of *Plasmodium **falciparum* Trans R Soc Trop Med Hyg 1971 65 549 559 10.1016/0035-9203(71)90036-8 5003557

[B3] Eichner M Diebner HH Molineaux L Collins WE Jeffery GM Dietz K Genesis, sequestration and survival of *Plasmodium **falciparum *gametocytes: parameter estimates from fitting a model to malariatherapy data Trans R Soc Trop Med Hyg 2001 95 497 501 10.1016/S0035-9203(01)90016-1 11706658

[B4] Pichon G Awono-Ambene HP Robert V High heterogeneity in the number of *Plasmodium **falciparum *gametocytes in the bloodmeal of mosquitoes fed on the same host Parasitol 2000 121 115 120 10.1017/S0031182099006277 11085230

[B5] Gaillard FO Boudin C Chau NP Robert V Pichon G Togetherness among *Plasmodium **falciparum *gametocytes: interpretation through simulation and consequences for malaria transmission Parasitol 2003 127 427 435 10.1017/S0031182003004025 14653532

[B6] Bachmann A Esser C Petter M Predehl S von Kalckreuth V Schmeidel S Bruchhaus I Tannich E Absence of erythrocyte sequestration and lack of multicopy gene family expression in *Plasmodium falciparum *from a splenectomized malaria patient PLoS One 2009 4 e7459 10.1371/journal.pone.0007459 19826486 PMC2758591

[B7] Day PD Hayward RE Smith D Cuvenor JG CD36-dependent adhesion and knob expression of the Transmission of *Plasmodium **falciparum *is stage-specific Mol Biochem Parasitol 1998 93 167 177 10.1016/S0166-6851(98)00040-1 9662702

[B8] Hayward RE Tiwari B Piper KP Baruch DI Day KP Virulence and Transmission success of the malaria parasite *Plasmodium **falciparum* Proc Natl Acad Sci USA 1999 96 4563 4568 10.1073/pnas.96.8.4563 10200302 PMC16372

[B9] Smith TG Serghides L Patel SN Febbraio M Silverstein RL Kain KC CD36-mediated nonopsonic phagocytosis of erythrocytes infected with stage I and IIA gametocytes of *Plasmodium **falciparum* Infect Immun 2003 71 393 400 10.1128/IAI.71.1.393-400.2003 12496189 PMC143147

[B10] Rogers NJ Hall BS Obiero J Targett GAT Sutherland CJ A model for sequestration of the transmission stages of *Plasmodium **falciparum*: adhesion of gametocyte-infected erythrocytes to human bone marrow cells Infect Immun 2000 68 3455 33462 10.1128/IAI.68.6.3455-3462.2000 10816498 PMC97624

[B11] Kaslow DC Transmission-blocking vaccines: uses and current status of development Int J Parasitol 1997 27 183 189 10.1016/S0020-7519(96)00148-8 9088989

[B12] Sutherland CJ Surface antigens of *Plasmodium falciparum *gametocytes - a new class of transmission-blocking vaccine targets? Mol Biochem Parasitol 2009 166 93 98 10.1016/j.molbiopara.2009.03.007 19450726

[B13] Sharp S Lavstsen T Fivelman QL Saeed M McRobert L Jensen AT Baker DA Theander TG Sutherland CJ Programmed transcription of the *var *gene family, but not of *stevor*, in *Plasmodium **falciparum *gametocytes Eukaryot Cell 2006 5 1206 1214 10.1128/EC.00029-06 16896206 PMC1539138

[B14] Kyes S Horrocks P Newbold C Antigenic variation at the infected red cell surface in malaria Annu Rev Microbiol 2001 55 673 707 10.1146/annurev.micro.55.1.673 11544371

[B15] Bull PC Lowe BS Kortok M Molyneux CS Newbold CI Marsh K Parasite antigens on the infected red cell surface are targets for naturally acquired immunity to malaria Nat Med 1998 4 358 360 10.1038/nm0398-358 9500614 PMC3836255

[B16] Bull PC Lowe BS Kortok M Marsh K Antibody recognition of *Plasmodium **falciparum *erythrocyte surface antigens in Kenya: evidence for rare and prevalent variants Infect Immun 1999 67 733 739 9916084 10.1128/iai.67.2.733-739.1999 PMC96380

[B17] Kraemer SM Smith JD Evidence for the importance of genetic structuring to the structural and functional specialization of the *Plasmodium falciparum var *gene family Mol Microbiol 2003 50 1527 1538 10.1046/j.1365-2958.2003.03814.x 14651636

[B18] Lavstsen T Salanti A Jensen ATR Arnot DE Theander TG Sub-grouping of *Plasmodium falciparum *3D7 *var *genes based on sequence analysis of coding and non-coding regions Malar J 2003 2 27 10.1186/1475-2875-2-27 14565852 PMC222925

[B19] Joannin N Abhiman S Sonnhammer EL Wahlgren M Sub-grouping and sub-functionalization of the RIFIN multi-copy protein family BMC Genomics 2008 9 19 10.1186/1471-2164-9-19 18197962 PMC2257938

[B20] Petter M Haeggström M Khattab A Fernandez V Klinkert MQ Wahlgren M Variant proteins of the *Plasmodium **falciparum *RIFIN family show distinct subcellular localization and developmental expression patterns Mol Biochem Parasitol 2007 156 51 61 10.1016/j.molbiopara.2007.07.011 17719658

[B21] Petter M Bonow I Klinkert MQ Diverse expression patterns of subgroups of the *rif *multigene family during *Plasmodium falciparum *gametocytogenesis PLoS One 2008 3 e3779 10.1371/journal.pone.0003779 19020666 PMC2582490

[B22] Ponnudurai T Lensen AH Meis JF Meuwissen JH Synchronization of *Plasmodium falciparum *gametocytes using an automated suspension culture system Parasitol 1986 93 263 274 10.1017/S003118200005143X 3537921

[B23] Lasonder E Janse CJ van Gemert GJ Mair GR Vermunt AM Douradinha BG van Noort V Huynen MA Luty AJ Kroeze H Khan SM Sauerwein RW Waters AP Mann M Stunnenberg HG Proteomic profiling of Plasmodium sporozoite maturation identifies new proteins essential for parasite development and infectivity PLoS Pathog 2008 4 e1000195 10.1371/journal.ppat.1000195 18974882 PMC2570797

[B24] Wang CW Magistrado PA Nielsen MA Theander TG Lavstsen T Preferential transcription of conserved rif genes in two phenotypically distinct *Plasmodium falciparum *parasite lines Int J Parasitol 2009 39 655 664 10.1016/j.ijpara.2008.11.014 19162031

[B25] Salanti A Staalsoe T Lavstsen T Jensen ATR Sowa MPK Arnot DE Hviid L Theander TG Selective upregulation of a single distinctly structured *var *gene in chondroitin sulphate A-adhering *Plasmodium **falciparum *involved in pregnancy-associated malaria Mol Microbiol 2003 49 179 191 10.1046/j.1365-2958.2003.03570.x 12823820

[B26] Dahlbäck M Lavstsen T Salanti A Hviid L Arnot DE Theander T Nielsen MA Changes in *var *gene mRNA levels during erythrocytic development in two phenotypically distinct *Plasmodium falciparum *parasites Malar J 2007 6 78 10.1186/1475-2875-6-78 17565661 PMC1904452

[B27] Jensen AT Magistrado P Sharp S Joergensen L Lavstsen T Chiucchiuini A Salanti A Vestergaard LS Lusingu JP Hermsen R Sauerwein R Christensen J Nielsen MA Hviid L Sutherland C Staalsoe T Theander TG *Plasmodium falciparum *associated with severe childhood malaria preferentially expresses PfEMP1 encoded by group A *var *genes J Exp Med 2004 199 1179 1190 10.1084/jem.20040274 15123742 PMC2211911

[B28] McRobert L Preiser P Sharp S Jarra W Kaviratne M Taylor MC Renia L Sutherland CJ Distinct trafficking and localization of STEVOR proteins in three stages of the *Plasmodium falciparum *life cycle Infect Immun 2004 72 6597 6602 10.1128/IAI.72.11.6597-6602.2004 15501792 PMC522994

